# Label Distribution Learning for Automatic Cancer Grading of Histopathological Images of Prostate Cancer

**DOI:** 10.3390/cancers15051535

**Published:** 2023-02-28

**Authors:** Mizuho Nishio, Hidetoshi Matsuo, Yasuhisa Kurata, Osamu Sugiyama, Koji Fujimoto

**Affiliations:** 1 Department of Radiology, Kobe University Graduate School of Medicine, 7-5-2 Kusunoki-cho, Chuo-ku, Kobe 650-0017, Japan; 2 Department of Diagnostic Imaging and Nuclear Medicine, Kyoto University Graduate School of Medicine, 54 Shogoin Kawahara-cho, Sakyo-ku, Kyoto 606-8507, Japan; 3 Department of Informatics, Kindai University, 3-4-1 Kowakae, Higashiosaka City 577-8502, Japan; 4 Department of Real World Data Research and Development, Kyoto University Graduate School of Medicine, 54 Shogoin Kawahara-cho, Sakyo-ku, Kyoto 606-8507, Japan

**Keywords:** prostate cancer, Gleason score, ISUP score, digital pathology, deep learning, label distribution learning

## Abstract

**Simple Summary:**

We aimed to develop and evaluate an automatic prediction system for grading histopathological images of prostate cancer using a deep learning model and label distribution learning. Our results show that the label distribution learning improved the diagnostic performance of the automatic prediction system for the cancer grading.

**Abstract:**

We aimed to develop and evaluate an automatic prediction system for grading histopathological images of prostate cancer. A total of 10,616 whole slide images (WSIs) of prostate tissue were used in this study. The WSIs from one institution (5160 WSIs) were used as the development set, while those from the other institution (5456 WSIs) were used as the unseen test set. Label distribution learning (LDL) was used to address a difference in label characteristics between the development and test sets. A combination of EfficientNet (a deep learning model) and LDL was utilized to develop an automatic prediction system. Quadratic weighted kappa (QWK) and accuracy in the test set were used as the evaluation metrics. The QWK and accuracy were compared between systems with and without LDL to evaluate the usefulness of LDL in system development. The QWK and accuracy were 0.364 and 0.407 in the systems with LDL and 0.240 and 0.247 in those without LDL, respectively. Thus, LDL improved the diagnostic performance of the automatic prediction system for the grading of histopathological images for cancer. By handling the difference in label characteristics using LDL, the diagnostic performance of the automatic prediction system could be improved for prostate cancer grading.

## 1. Introduction

In 2022, 34,500 Americans were predicted to die of prostate cancer [1]. Prostate cancer is the second leading cause of cancer-related death in men. It is also the leading cause of cancer-related morbidity in men, with 268,490 cases [1]. Although the 5-year survival rate of prostate cancer has improved in recent years, the number of deaths is considerably high due to the large number of patients with prostate cancer. Bone metastases from prostate cancer frequently occur and reduce the patients’ quality of life.

Prostate cancer is diagnosed by palpation, prostate-specific antigen testing, ultrasound examination, magnetic resonance imaging, and biopsy. The definitive diagnosis of prostate cancer is made by performing a pathological evaluation of the prostate tissue obtained through biopsy or surgery. A visual evaluation score called the Gleason score is used for pathological evaluation of prostate tissues [2,3]. It is determined based on the classification system of histological morphology with five-class patterns. For each specimen, the pattern with the largest area is regarded as the first pattern, while the pattern with the second largest area is regarded as the second pattern. The sum of the two patterns is then calculated to obtain the Gleason score. For example, if the values of the first and second patterns are 4 and 5, respectively, the Gleason score is 9 (4 + 5).

Various problems associated with the determination of the Gleason score have been identified. Hence, the International Society of Urological Pathology (ISUP) proposed a new grading system, called the ISUP grade group classification system (ISUP score), based on the Gleason score [3]. The appropriate ISUP score was assigned by grouping the Gleason scores, with higher ISUP scores indicating higher malignancy.

A previous study evaluated the reproducibility of the Gleason score and its interobserver variability [4,5,6]. One previous study showed moderate agreement on the Gleason score between the observers [4]. As the ISUP score is based on the Gleason score, the agreement on the ISUP score is expected to be moderate.

The recent emergence of deep learning provides powerful tools for medical image analysis [7]. Deep learning makes it possible to extract valuable features in an end-to-end manner. Many studies have used deep learning for medical image analysis in radiology [7,8,9,10] and pathology [11,12,13,14,15]. To improve the inter-observer variability of the Gleason and ISUP scores, deep learning has been used for automated grading systems [15,16,17,18,19,20,21,22,23]. Table 1 presents a summary of the results of previous studies on automatic prediction systems for Gleason and ISUP scores. Table 1 shows that the size of the dataset used in the deep learning-based systems of previous studies was less than 10,000. As shown in the previous study [10], it is difficult to construct reliable deep-learning-based systems with small-sized datasets.

This study aimed to (i) use a large dataset for the development of a deep-learning-based ISUP grading system, (ii) evaluate the generalizability of the automated ISUP grading, and (iii) incorporate label distribution learning (LDL) [24,25,26] in system development with deep learning to address a difference in label characteristics between datasets. Of these, the primary goal of the current study is to combine deep learning with LDL and evaluate the utility of LDL in the ISUP grading. 

**Table 1:** Summary of previous studies on the automatic prediction systems of Gleason or ISUP scores.

Authors	Origin of Dataset or Dataset Name	Size of Dataset	Diagnostic Performance of Systems	Comment
Nagpal et al. [22]	TCGA Dataset Naval Medical Center San Diego Marin Medical Laboratories	1226 slides for training 331 slides for validation	70% accuracy on the Gleason scoring task	DL
Arvaniti et al. [27]	University Hospital Zurich	641 patients for training 245 patients for testing	Cohen’s quadratic kappa was evaluated the inter-pathologist agreement (kappa = 0.71). kappa = 0.75 between DL and pathologist1 and kappa = 0.71 between DL and pathologist2.	DL 6-class classification based on the Gleason score.
Lucas et al. [20]	Amsterdam University Medical Centers	96 tissue sections from 38 patients	Concordance of adjusted grade groups between the automated determination method and a genitourinary pathologist was obtained in 65% (A quadratic weighted kappa = 0.70)	DL 4-class classification based on the Gleason score.
Bulten et al. [19]	Radboud University Medical Center	5759 biopsies from 1243 patients	In an observer experiment, the deep learning system scored higher (kappa 0.854) than the panel (median kappa 0.819), outperforming 10 of 15 pathologist observers.	DL 6-class classification based on the Gleason score.
Egevad et al. [18]	Pathology Imagebase dataset hosted on the ISUP Web site	87 needle biopsies	The mean weighted kappas of panel members for all cases, the consensus cases, and the non-consensus cases were 0.67, 0.77, and 0.50, respectively. The weighted kappas of the AI system against the observers for all cases, the consensus cases, and the non-consensus cases were 0.63, 0.66, and 0.53, respectively.	DL developed in the previous study All cases were graded by 23 panel members. 5-class classification The experts failed to reach a 2/3 consensus in 41.4% (36/87).
Kwak et al. [16]	National Institutes of Health.	73 benign and 89 cancer samples for training 217 benign and 274 cancer samples for testing	AUC = 0.974	DL Binary classification (benign/cancer) Four tissue microarrays (TMAs) were used.
Singhal et al. [15]	PANDA challenge dataset (Radboud University Medical Center and Karolinska Institute) Muljibhai Patel Urological Hospital (MPUH)	580 biopsies from MPUH. The dataset was split into training (155) and testing sets (425). 3586 biopsies from Radboud University Medical Center for training and 1201 for testing. 1303 biopsies from the Karolinska Institute for unseen test data.	accuracy of 83.1% and a quadratic weighted kappa of 0.93 for the 1303 biopsies of unseen test data.	DL Part of PANDA challenge dataset was used. 6-class classification (5 classes of IUSP + benign)

Abbreviations: DL, deep learning.

## 2. Materials and Methods

### 2.1. Dataset

A total of 10,616 whole slide images (WSIs) were used in this study, which are available from the Prostate cANcer graDe Assessment (PANDA) challenge [14,28]. The 5160 and 5456 WSIs of the PANDA dataset were collected from Radboud University Medical Center and Karolinska Institute, respectively. A summary of the PANDA dataset is presented in Table 2. The details of the PANDA dataset are described in the published paper [14] and the Kaggle website [29]. 

The two institutions used different scanners with slightly different maximum microscopic resolutions. The annotation process for the 5160 and 5456 WSIs differed between the two institutions. At the Radboud University Medical Center, labels of the 5160 WSIs were retrieved from pathology reports, containing the original diagnosis. Trained students read all the reports and assigned a label to each WSI. At the Karolinska Institute, the 5456 WSIs were annotated by an experienced pathologist. Therefore, the labels of 5160 WSIs from Radboud University Medical Center might have a higher degree of inconsistency than those from the Karolinska Institute. In addition, Table 2 shows that the frequencies of ISUP scores were different between Radboud University Medical Center and Karolinska Institute; the frequencies of ISUP scores were more uniform at Radbound University Medical Center than at Karolinska Institute.

To develop and evaluate our deep learning-based systems, 5160 WSIs from Radboud University Medical Center and 5456 WSIs from Karolinska Institute were used as the development (training/validation sets) and unseen test sets, respectively. Table 2 shows that there was a difference in the frequency of ISUP scores between Radboud University Medical Center and Karolinska Institute. In addition, the 5160 WSIs of Radboud University Medical Center, which might have higher label inconsistency, were used for the development of a deep learning-based system. Therefore, it was expected that the dataset splitting of the current study would cause a deterioration in diagnostic performance compared with that of the original PANDA challenge.

### 2.2. Baseline Convolutional Neural Network

**Figure 1:**
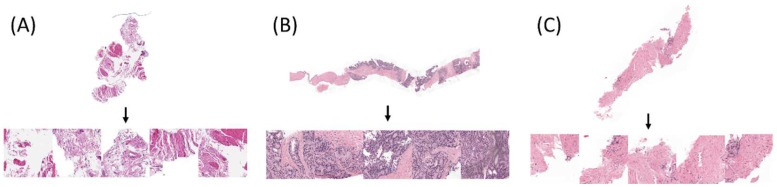
Original WSIs and their tiled images with different ISUP scores ((**A**) ISUP score = 0, (**B**) ISUP score = 4, and (**C**) ISUP score = 5). Note: For brevity, only five tiled images are shown. (**A**) ISUP score = 0 and Gleason score = 0 + 0 at the Karolinska Institute; (**B**) ISUP score = 4 and Gleason score = 4 + 4 at Radboud University Medical Center; (**C**) ISUP score = 5 and Gleason score = 4 + 5 at Radboud University Medical Center. Abbreviations: whole slide image (WSI).

In the PANDA challenge, many deep learning-based systems have been developed using convolutional neural networks (CNNs). After the PANDA challenge, the source codes of several CNNs were made available as open sources. For example, the source code of the first-place solution used in the PANDA challenge can be obtained from the GitHub repository [30]. Their CNN ranked 22nd (metric = 0.910) and 1st (metric = 0.940) on the public and private leaderboards of the PANDA challenge, respectively. The details of the first-place solutions are described on their slide [31]. The first solution consists of several types of CNNs. One of the CNNs of the first-place solution was used as the baseline CNN in this study. To implement our baseline CNN (network structure, image preprocessing, loss function, etc.), the source code of the first-place solution was used. Based on the first-place solution, our baseline CNN used EfficientNet [32] (EfficientNet B1) for the network structure, which was pretrained with the ImageNet dataset. Because the original WSIs contained non-tissue lesions, the input images of the baseline CNN were preprocessed by creating tiled images as the first-place solution. In image tiling, the original WSIs were split into several tiles (image patches), tiles with non-tissue lesions were removed, and the tiles were concatenated as the input image. Figure 1 shows representative images of the original WSIs and their tiles. After image tiling, the original WSI was converted into a tiled image of 1536 × 1536 × 3 (1536 = 8 tile × 192) in size. Based on the first-place solution, the labels of the WSIs in our baseline CNN were represented as 10-dimensional vectors, where the first and second five dimensions were derived based on the ISUP score and the first pattern of the Gleason score, respectively. For example, the ISUP score and the first pattern of the Gleason score were 3 and 4, respectively, and the scores 3 and 4 were converted to [1, 1, 1, 0, 0] and [1, 1, 1, 1, 0], respectively. Then, the [1, 1, 1, 0, 0] and [1, 1, 1, 1, 0] were concatenated as [1, 1, 1, 0, 0, 1, 1, 1, 1, 0]. The [1, 1, 1, 0, 0, 1, 1, 1, 1, 0] was used as the label of the WSI for our baseline CNN. The loss function of our baseline CNN was regarded as the binary cross-entropy loss between the 10-dimensional vectors of the outputs and labels.

### 2.3. Proposed CNN with LDL

The primary goal of the current study is to combine deep learning with LDL [24,25,26]. For this purpose, LDL was incorporated into our proposed CNN. Previous studies suggested that LDL could be used to address label inconsistency issues in traditional single labels. Instead of assigning a single label, LDL covers a certain number of adjacent labels, where each label represents a different degree of description. Given the N input training WSIs with the corresponding single labels of ISUP scores and the first pattern of Gleason scores, the dataset is represented as { (x_1_, y_1_, z_1_), ……, (x_N_, y_N_, z_N_) }, where x_i_ represents WSI, y_i_ represents the ISUP score of WSI (y_i_ ∈ [0, ……, 5]), and z_i_ represents the first pattern of the Gleason score (z_i_ ∈ [0, ……, 5]). For WSI x_i_, the label distribution $d_{i}^{c}$ (c = 1, 2, 3, …, D, where D is the maximum number of label distributions) was generated based on the following Gaussian function:

(1) $$d_{i}^{c}=\frac{1}{\sqrt{2\pi }\sigma }exp\left(\frac{-{\left(c-l_{i}\right)}^{2}}{2\sigma^{2}}\right),$$

where *l_i_* is the rescaled value of *y_i_* or *z_i_* and σ  is the standard deviation of the Gaussian function. In this study, the values of *l_i_* were obtained from *y_i_* or *z_i_* using linear scaling based on the value of *D* (range of *l_i_* matched with [1, …, *D*]). $d_{i}^{c}$ represents the description degree (probability) of a label. As the maximum of the Gaussian function was obtained at *c* = *l_i_*, the probability at *l_i_* was the highest. However, the probabilities at $c=l_{i}-1\;$ or $c=l_{i}+1$ were not 0 in general. Based on the label distribution, label inconsistency caused by label noise was handled in the LDL. The raw values of $d_{i}^{c}$ were normalized to satisfy the following conditions: (i) $d_{i}^{c}$ ∈ [0, 1] and (ii) $\overset{D}{\underset{c=1}{\sum }}d_{i}^{c}$. Representative examples of label distributions are shown in Supplementary Material S1 (Figure S1A–D).

The network structure of our proposed CNN with LDL was based on EfficientNet. In our proposed CNN, the base part with convolutional layers of EfficientNet was retained, while the head part of EfficientNet was replaced with three fully connected layers with the following numbers of outputs: 512, 100, and 2D. The three layers accompanied the batch normalization and activation layers of the rectified linear unit. Our proposed CNN received tiled WSIs and generated a 2D-dimensional vector. The first and second D-dimensional vector ($o_{i}^{1}$ and $o_{i}^{2}$) outputs from our proposed CNN corresponded to two vectors of $d_{i}^{c}$ obtained from y_i_ and z_i_. Training of our proposed CNN was accomplished by minimizing the KL divergence between the predicted and ground-truth label distributions, which is represented by the following equation:

(2) $${\mathrm{KL}}_{\mathrm{div}}(o_{i}^{1},\;d_{i}^{c}\_from\_y_{i})\;+\mathrm{weight}\;\times \;{\mathrm{KL}}_{\mathrm{div}}(o_{i}^{2},\;d_{i}^{c}\_from\_z_{i})$$

where $d_{i}^{c}\_from\_y_{i}$ and $d_{i}^{c}\_from\_z_{i}$ were obtained from *y_i_* and *z_i_*, respectively, with Equation (1) showing the label distributions. Here, *KL_div_* is the KL divergence between two probability distributions.

### 2.4. Implementation Details

PyTorch (version 1.9.0) and PyTorch Lightning (version 1.6.0) were used as the deep learning frameworks in this study. The baseline CNN was implemented based on the open-source code of the first-place solution in the PANDA challenge. As the original source code of the first-place solution used the old version of PyTorch Lightning (version 0.8.5), the source code for our baseline CNN was revised using the new version of PyTorch Lightning. The hyperparameters of our baseline CNN are available from https://github.com/kentaroy47/Kaggle-PANDA-1st-place-solution/blob/master/src/configs/final_1.yaml (accessed on 6 January 2023). Briefly, the hyperparameters of our baseline CNN were as follows: number of epochs, 30; optimizer, Adam; learning rate, 3.0 × 10^−5^; and size of input (tiled image), 1536 × 1536 × 3. The proposed CNN with LDL was implemented by modifying the source code of the baseline CNN. The base part of the proposed CNN was a pretrained EfficientNet (B0–B5) model. The hyperparameters specific to the proposed CNN were as follows: *weight* of LDL = 0.20 (Equation (2)), σ=2.0, and D = 18.

### 2.5. Evaluation of CNNs

In the baseline CNN, the predicted label was obtained by summing the first 5-dimensional vector of its output. This method was implemented in the original source code of the first solution. In the proposed CNN with LDL, the raw predicted label of the proposed CNN was determined using the following equation:

(3) $$pl_{i}=argmax\left(o_{i}^{1}\right),$$

where $pl_{i}\;$ is the predicted raw label of the proposed CNN. Finally, the value of $pl_{i}\;$ was inverted via linear rescaling based on the value of *D*, and the final predicted label for the proposed CNN was obtained. In the baseline CNN and proposed CNN, the predicted Gleason score was not used for evaluation. Five-fold cross-validation was performed for the baseline CNN and proposed CNN using the development set (5160 WSIs from Radboud University Medical Center). After the five-fold cross-validation, the unseen test set (5456 WSIs from the Karolinska Institute) was used to evaluate the CNNs. The prediction results for the test set were obtained using an ensemble of five trained models for the baseline and proposed CNNs. As the whole process of prediction is not available in the source code of the first-place solution, it was implemented for the baseline and proposed CNNs in this study.

The evaluation metrics used in this study were quadratic weighted kappa (QWK) and accuracy. QWK, the main metric utilized in the PANDA challenge, was used to evaluate the agreement between the ground truth label and predicted label. The QWK typically varies from 0 (random agreement) to 1 (complete agreement). QWK was calculated as follows: First, a confusion matrix *M* was constructed from the ground truth and predicted labels. The size of *M* was *N*-by-*N* (*N* = 6, range of ground truth label; 0–5, range of predicted labels). In matrix *M*, *M_i,j_* corresponded to the number of ISUP scores *i* (ground truth) that received a predicted value *j*. The *N*-by-*N* matrix of weights *w* was calculated based on the difference between the actual and predicted values using the following equation:

(4) $$w_{i,j}=\frac{{\left(i-j\right)}^{2}}{{\left(N-1\right)}^{2}}.$$

The *N*-by-*N* confusion matrix of the expected outcomes, *E*, was calculated assuming that there was no correlation between the ground truth and predicted labels. In all three matrices (*M*, *w*, and *E*), QWK was calculated as follows:

(5) $$\mathrm{QWK}=\frac{\sum_{i,j}w_{i,j}M_{i,j}}{\sum_{i,j}w_{i,j}E_{i,j}}.$$

Accuracy was defined as the ratio of diagonal elements in matrix *M* calculated between the ground truth and predicted labels. In this study, the IUSP scores were used as the ground truth in the calculation of QWK and accuracy; the Gleason scores were ignored when calculating the evaluation metrics of this study.

To statistically test the difference in accuracy between the baseline and proposed CNNs, McNemar’s test was used. A *p*-value < 0.05 was considered to indicate statistical significance.

## 3. Results

Table 3 presents the results of five-fold cross-validation of the baseline and proposed CNNs (EfficientNet B0–5) using the development set. The cross-validated QWK and the accuracy of the baseline CNN were 0.820 and 0.545, respectively. The cross-validated QWK and accuracy of the proposed CNNs were 0.817–850 and 0.646–0.680, respectively. In most cases, the cross-validated QWK and accuracy of the proposed CNNs were better than those of the baseline CNN. The proposed CNN of EfficientNet B3 was the best, as shown in Table 3.

Table 4 shows the results of five-fold cross-validation of the proposed CNNs with different *D* values (EfficientNet B3 only). Here, *D* = 12, 30, and 60 were used in addition to *D* = 18. The cross-validated QWK and accuracy of the proposed CNNs were 0.835–850 and 0.609–0.700, respectively (Table 4). The proposed CNN with *D* = 60 achieved the highest accuracy (Table 4).

The cross-validated QWK and accuracy of the proposed CNN of EfficientNet B3 with LDL (*D* = 18) and another proposed CNN of EfficientNet B3 with LDL (*D* = 60) were relatively high (Table 3 and Table 4, respectively). Hence, these two types of proposed CNNs were evaluated using the test set.

**Figure 2:**
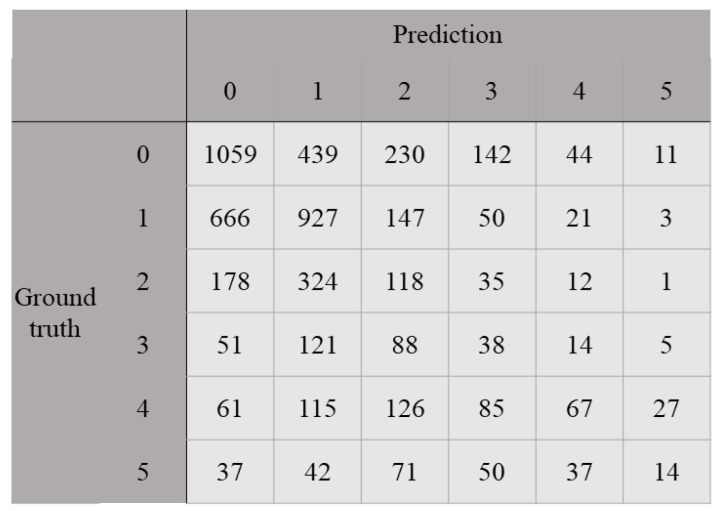
Confusion matrix of the proposed CNN between the ground truth and predicted labels in the test set. Note: EfficientNet B3 with LDL (*D* = 60) was used.

Table 5 shows the diagnostic performance of the baseline and proposed CNNs in the unseen test set. The QWK and accuracy of the baseline CNN were 0.240 and 0.247, respectively, in the test set. The QWK and accuracy of the two types of proposed CNNs were 0.301 and 0.364, and 0.249 and 0.407, respectively, in the test set. The difference in accuracy between the baseline CNN and the proposed CNN (EfficientNet B3 with LDL (*D* = 60)) was statistically significant (*p*-value < 0.000001). As a result, the proposed CNNs can improve the diagnostic performance of the first-place solution for the automatic prediction of prostate cancer grade. Figure 2 shows the confusion matrix between the ground truth and the predicted label for the proposed CNN of EfficientNet B3 with LDL (*D* = 60). 

Table 3, Table 4 and Table 5 show that when the development set (5160 WSIs from Radboud University Medical Center) was used, the diagnostic performance of the baseline and proposed CNNs severely deteriorated in the test set (5456 WSIs from the Karolinska Institute).

## 4. Discussion

Table 5 shows that the proposed CNNs could achieve better diagnostic performance for the automatic prediction of ISUP scores compared with the baseline CNN. Because the major difference between the proposed CNNs and the baseline CNNs was the use of LDL, LDL was useful for improving the CNNs and predicting the ISUP scores automatically. However, for the proposed CNN and baseline CNN, their diagnostic performance in the test set was worse than that in the development set.

As previously mentioned in Materials and Methods section and Table 2, the labels of 5160 WSIs from the Radboud University Medical Center were annotated by trained students. Therefore, these labels might have a higher degree of inconsistency compared with those from the Karolinska Institute. The label inconsistency of WSIs in the Radboud University Medical Center might decrease the generalizability of the model in the development of the proposed and baseline CNNs. In addition, the difference in label frequency between Radboud University Medical Center and Karolinska Institute might deteriorate the model’s generalizability. Our results and speculation indicate that when a large number of images with low-quality labels are available, the development of a deep learning model may lead to the deterioration of the generalizability of the model. This point should be carefully considered when developing deep-learning-based systems.

Issues related to label inconsistency have been reported in the original PANDA challenge. However, the discrepancy in diagnostic performance between the development and test sets was not extremely large during the original PANDA challenge because the development set consisted of WSIs obtained from the two institutions. In this study, the discrepancy in diagnostic performance between the development and test sets was significantly large because the development set was derived from one institution. 

LDL has been proposed to address the issues related to label inconsistency on traditional single labels. In this study, the diagnostic performance of our system could be improved by incorporating LDL into the deep learning-based system. Owing to the characteristics of LDL, it might be useful for other medical systems to resolve label inconsistency issues in the grading scores (cancer staging, grading for cancer diagnosis, and so on).

Table 1 presents a summary of the results of previous studies on automatic prediction systems for Gleason and ISUP scores. The size of the dataset in this study was larger than that in the previous studies. However, the diagnostic performance of our CNNs was worse than that of the CNNs in the previous studies. As shown, the differences in label inconsistency and label frequency between Radbound University Medical Center and Karolinska Institute may cause the performance discrepancy.

This study has several limitations. First, it was conducted using a public dataset (the PANDA dataset). Our results should be confirmed using other datasets. Second, our results were obtained using EfficientNet. Although EfficientNet is a standard deep learning model, other deep learning models should be used to evaluate the usefulness of LDL. As the first-place solution to the PANDA challenge used EfficientNet in the source code, EfficientNet was also used in this study. 

## 5. Conclusions

Our proposed CNN with LDL could improve the cancer grading (ISUP scores) of histopathological images in prostate cancer. This improvement could be achieved with the aid of LDL (a strategy used to address label inconsistency issues). However, when the difference in label characteristics (label inconsistency and label frequency) between the development and test sets was observed, the generalizability of the baseline and proposed CNNs deteriorated in the unseen test set. Our results should be carefully considered when developing deep-learning-based systems.

## Figures and Tables

**Table 2:** Summary of the PANDA dataset.

KERRYPNX	Radboud University Medical Center	Karolinska Institute
Number of WSIs	N = 5160	N = 5456
Frequency of ISUP scores	ISUP score 0, N = 967	ISUP score 0, N = 1925
	ISUP score 1, N = 852	ISUP score 1, N = 1814
	ISUP score 2, N = 675	ISUP score 2, N = 668
	ISUP score 3, N = 925	ISUP score 3, N = 317
	ISUP score 4, N = 768	ISUP score 4, N = 481
	ISUP score 5, N = 973	ISUP score 5, N = 251
Annotators	trained students	a single experienced pathologist
Usage in this study	development set (training/validation sets)	unseen test set

Abbreviations: Prostate cANcer graDe Assessment (PANDA), whole slide image (WSI), and the International Society of Urological Pathology (ISUP).

**Table 3:** Results of the five-fold cross-validation of the baseline and proposed CNNs (EfficientNet B0–5).

CNN	Cross-Validated QWK	Cross-Validated Accuracy
Baseline CNN	0.820	0.545
Proposed CNN of EfficientNet B0 with LDL	0.817	0.646
Proposed CNN of EfficientNet B1 with LDL	0.836	0.663
Proposed CNN of EfficientNet B2 with LDL	0.840	0.667
Proposed CNN of EfficientNet B3 with LDL	0.850	0.680
Proposed CNN of EfficientNet B4 with LDL	0.840	0.663
Proposed CNN of EfficientNet B5 with LDL	0.832	0.654

Note: The development set (5160 WSIs from Radboud University Medical Center) was used for the five-fold cross validation. In the proposed CNN, the following parameters were used: the weight for LDL = 0.20, 𝜎 = 2.0, and *D* = 18. Abbreviations: WSI, whole slide image; CNN, convolutional neural network; QWK, quadratic weighted kappa; LDL, label distribution learning.

**Table 4:** Results of the five-fold cross validation of the proposed CNNs with different *D* values (EfficientNet B3 only).

CNN	Cross-Validated QWK	Cross-Validated Accuracy
Proposed CNN with LDL (*D* = 18)	0.850	0.680
Proposed CNN with LDL (*D* = 12)	0.842	0.609
Proposed CNN with LDL (*D* = 30)	0.835	0.683
Proposed CNN with LDL (*D* = 60)	0.839	0.700

Note: The development set (5160 WSIs from Radboud University Medical Center) was used for the five-fold cross validation. Abbreviations: WSI, whole-slide image; CNN, convolutional neural network; QWK, quadratic weighted kappa.

**Table 5:** Performance of the baseline and proposed CNNs in the unseen test set.

CNN	QWK	Accuracy
Baseline CNN	0.240	0.247
Proposed CNN of EfficientNet B3 with LDL (*D* = 18)	0.301	0.249
Proposed CNN of EfficientNet B3 with LDL (*D* = 60)	0.364	0.407

Note: The two types of proposed CNNs were used in the evaluation of the unseen test set as their cross-validated QWK and accuracy were relatively high. The test set (5456 WSIs from the Karolinska Institute) is used in Table 5. Abbreviations: WSI, whole slide image; CNN, convolutional neural network; LDL, label distribution learning; QWK, quadratic weighted kappa.

## Data Availability

The public dataset is available on the Kaggle website. The data presented in this study are available on request from the corresponding author.

## Supplementary Materials

The following supporting information can be downloaded at: https://www.mdpi.com/article/10.3390/cancers15051535/s1, Material S1 (including Figure S1A–D), Example of label distribution; Material S2 (including Table S2): Results of the five-fold cross validation (CV) for the baseline CNN and the proposed CNN.

## References

[1] Siegel R.L., Miller K.D., Fuchs H.E., Jemal A. (2022). Cancer statistics, 2022. CA Cancer J. Clin..

[2] Gleason D.F. (1992). Histologic grading of prostate cancer: A perspective. Hum. Pathol..

[3] Epstein J.I., Egevad L., Amin M.B., Delahunt B., Srigley J.R., Humphrey P.A. (2016). The 2014 international society of urological pathology (ISUP) consensus conference on gleason grading of prostatic carcinoma definition of grading patterns and proposal for a new grading system. Am. J. Surg. Pathol..

[4] Ozkan T.A., Eruyar A.T., Cebeci O.O., Memik O., Ozcan L., Kuskonmaz I. (2016). Interobserver variability in Gleason histological grading of prostate cancer. Scand. J. Urol..

[5] Allsbrook W.C., Mangold K.A., Johnson M.H., Lane R.B., Lane C.G., Epstein J.I. (2001). Interobserver reproducibility of Gleason grading of prostatic carcinoma: General pathologist. Hum. Pathol..

[6] Di Loreto C., Fitzpatrick B., Underhill S., Kim D.H., Dytch H.E., Galera-Davidson H., Bibbo M. (1991). Correlation Between Visual Clues, Objective Architectural Features, and Interobserver Agreement in Prostate Cancer. Am. J. Clin. Pathol..

[7] Yamashita R., Nishio M., Do R.K.G., Togashi K. (2018). Convolutional neural networks: An overview and application in radiology. Insights Imaging.

[8] Moribata Y., Kurata Y., Nishio M., Kido A., Otani S., Himoto Y., Nishio N., Furuta A., Onishi H., Masui K. (2023). Automatic segmentation of bladder cancer on MRI using a convolutional neural network and reproducibility of radiomics features: A two-center study. Sci. Rep..

[9] Noguchi S., Nishio M., Sakamoto R., Yakami M., Fujimoto K., Emoto Y., Kubo T., Iizuka Y., Nakagomi K., Miyasa K. (2022). Deep learning-based algorithm improved radiologists’ performance in bone metastases detection on CT. Eur. Radiol..

[10] Matsuo H., Nishio M., Kanda T., Kojita Y., Kono A.K., Hori M., Teshima M., Otsuki N., Nibu K.-i, Murakami T. (2020). Diagnostic accuracy of deep-learning with anomaly detection for a small amount of imbalanced data: Discriminating malignant parotid tumors in MRI. Sci. Rep..

[11] Steiner D.F., Macdonald R., Liu Y., Truszkowski P., Hipp J.D., Gammage C., Thng F., Peng L., Stumpe M.C. (2018). Impact of Deep Learning Assistance on the Histopathologic Review of Lymph Nodes for Metastatic Breast Cancer. Am. J. Surg. Pathol..

[12] Woerl A.C., Eckstein M., Geiger J., Wagner D.C., Daher T., Stenzel P., Fernandez A., Hartmann A., Wand M., Roth W. (2020). Deep Learning Predicts Molecular Subtype of Muscle-invasive Bladder Cancer from Conventional Histopathological Slides. Eur. Urol..

[13] Wei J.W., Tafe L.J., Linnik Y.A., Vaickus L.J., Tomita N., Hassanpour S. (2019). Pathologist-level classification of histologic patterns on resected lung adenocarcinoma slides with deep neural networks. Sci. Rep..

[14] Bulten W., Kartasalo K., Chen P.H.C., Ström P., Pinckaers H., Nagpal K., Cai Y., Steiner D.F., van Boven H., Vink R. (2022). Artificial intelligence for diagnosis and Gleason grading of prostate cancer: The PANDA challenge. Nat. Med..

[15] Singhal N., Soni S., Bonthu S., Chattopadhyay N., Samanta P., Joshi U., Jojera A., Chharchhodawala T., Agarwal A., Desai M. (2022). A deep learning system for prostate cancer diagnosis and grading in whole slide images of core needle biopsies. Sci. Rep..

[16] Kwak J.T., Hewitt S.M. (2017). Nuclear Architecture Analysis of Prostate Cancer via Convolutional Neural Networks. IEEE Access.

[17] Ren J., Sadimin E., Foran D.J., Qi X. Computer aided analysis of prostate histopathology images to support a refined Gleason grading system. Proceedings of the Medical Imaging 2017, Image Processing, SPIE.

[18] Egevad L., Swanberg D., Delahunt B., Ström P., Kartasalo K., Olsson H., Berney D.M., Bostwick D.G., Evans A.J., Humphrey P.A. (2020). Identification of areas of grading difficulties in prostate cancer and comparison with artificial intelligence assisted grading. Virchows Arch..

[19] Bulten W., Pinckaers H., van Boven H., Vink R., de Bel T., van Ginneken B., van der Laak J., Hulsbergen-van de Kaa C., Litjens G. (2020). Automated deep-learning system for Gleason grading of prostate cancer using biopsies: A diagnostic study. Lancet Oncol..

[20] Lucas M., Jansen I., Savci-Heijink C.D., Meijer S.L., de Boer O.J., van Leeuwen T.G., de Bruin D.M., Marquering H.A. (2019). Deep learning for automatic Gleason pattern classification for grade group determination of prostate biopsies. Virchows Arch..

[21] Jiménez del Toro O., Atzori M., Otálora S., Andersson M., Eurén K., Hedlund M., Rönnquist P., Müller H. Convolutional neural networks for an automatic classification of prostate tissue slides with high-grade Gleason score. Proceedings of the Medical Imaging 2017, Digital Pathology, SPIE.

[22] Nagpal K., Foote D., Liu Y., Chen P.H.C., Wulczyn E., Tan F., Olson N., Smith J.L., Mohtashamian A., Wren J.H. (2019). Development and validation of a deep learning algorithm for improving Gleason scoring of prostate cancer. NPJ Digit. Med..

[23] Linkon A.H.M., Labib M.M., Hasan T., Hossain M., Jannat M.E. (2021). Deep Learning in Prostate Cancer Diagnosis and Gleason Grading in Histopathology Images: An Extensive Study. Informatics in Medicine Unlocked.

[24] Geng X., Yin C., Zhou Z.H. (2013). Facial age estimation by learning from label distributions. IEEE Trans. Pattern Anal. Mach. Intell..

[25] Luo J., He B., Ou Y., Li B., Wang K. (2021). Topic-based label distribution learning to exploit label ambiguity for scene classification. Neural Comput. Appl..

[26] Wu X., Wen N., Liang J., Lai Y.K., She D., Cheng M.M., Yang J. (2019). Joint acne image grading and counting via label distribution learning. Proceedings of the IEEE International Conference on Computer Vision.

[27] Arvaniti E., Fricker K.S., Moret M., Rupp N., Hermanns T., Fankhauser C., Wey N., Wild P.J., Rüschoff J.H., Claassen M. (2018). Automated Gleason grading of prostate cancer tissue microarrays via deep learning. Sci. Rep..

[28] Bulten W., Litjens G., Pinckaers H., Ström P., Eklund M., Kartasalo K., Demkin M., Dane S. The PANDA challenge: Prostate cANcer graDe Assessment using the Gleason grading system. Proceedings of the 23rd International Conference on Medical Image Computing and Computer Assisted Intervention (MICCAI 2020).

[29] Prostate cANcer graDe Assessment (PANDA) Challenge | Kaggle. https://www.kaggle.com/c/prostate-cancer-grade-assessment.

[30] GitHub—Kentaroy47/Kaggle-PANDA-1st-Place-Solution: 1st Place Solution for the Kaggle PANDA Challenge. https://github.com/kentaroy47/Kaggle-PANDA-1st-place-solution.

[31] RistKaggleWorkshop_20200924_PANDA_1st—Google Slide. https://docs.google.com/presentation/d/1Ies4vnyVtW5U3XNDr_fom43ZJDIodu1SV6DSK8di6fs/edit#slide=id.p.

[32] Tan M., Le Q.V. EfficientNet: Rethinking model scaling for convolutional neural networks. Proceedings of the 36th International Conference of Machine Learning PMLR 2019.

