# Supplementary material for: Label Distribution Learning for Automatic Cancer Grading of Histopathological Images of Prostate Cancer
Source: Cancers (Basel). 2023 Feb 28;15(5):1535. doi: 10.3390/cancers15051535 (PMC10000939; doi:10.3390/cancers15051535)
Supplement: Supplementary file 1 [file cancers-15-01535-s001.zip › cancers-2248015-supplementary.pdf]

Supplementary Material S1

Example of the label distribution

For an example, we assume that the ISUP score and the first pattern of Gleason score are y_i = 3 and z_i = 4, respectively. In this case, our baseline CNN uses the labels = [1, 1, 1, 0, 0] for y_i = 3 and [1, 1, 1, 1, 0] for z_i = 4; the proposed CNN with LDL uses the label distribution = [7.43x10^{-7}, 7.99x10^{-6}, 6.69x10^{-5}, 4.36x10^{-4}, 2.22x10^{-3}, 8.76x10^{-3}, 2.70x10^{-2}, 6.48x10^{-2}, 1.21x10^{-1}, 1.76x10^{-1}, 1.99x10^{-1}, 1.76x10^{-1}, 1.21x10^{-1}, 6.48x10^{-2}, 2.70x10^{-2}, 8.76x10^{-3}, 2.22x10^{-3}, 4.36x10^{-4}] and l_i = 10 for y_i = 3, and the label distribution = [1.35x10^{-10}, 3.07x10^{-9}, 5.45x10^{-8}, 7.52x10^{-7}, 8.08x10^{-6}, 6.77x10^{-5}, 4.41x10^{-4}, 2.24x10^{-3}, 8.87x10^{-3}, 2.73x10^{-2}, 6.55x10^{-2}, 1.22x10^{-1}, 1.78x10^{-1}, 2.01x10^{-1}, 1.78x10^{-1}, 1.22x10^{-1}, 6.55x10^{-2}, 2.73x10^{-2}] and l_i = 13 for z_i = 4 when σ = 2.0 and D = 18. Figures S1A and S1B shows the graph representation of the labels for our baseline CNN. Figures S1C and S1D shows the graph representation of the label distributions for our proposed CNN with LDL.

Figure S1A

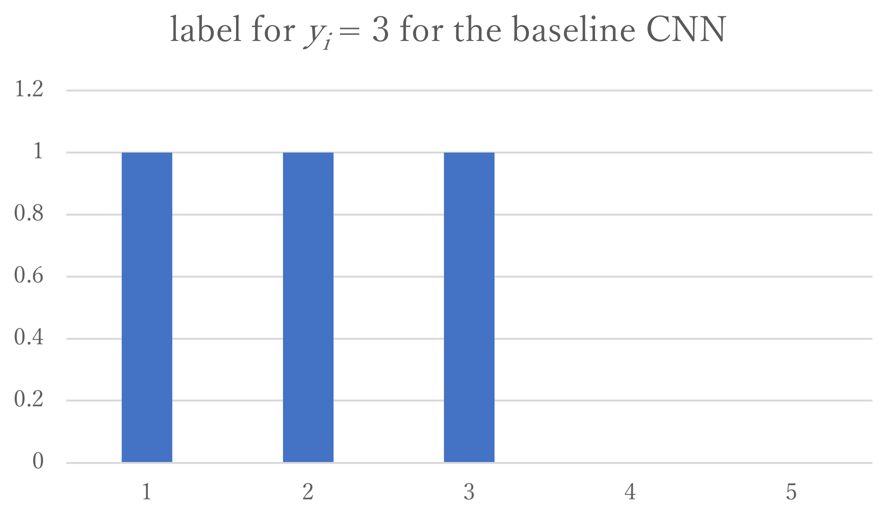

Figure S1B

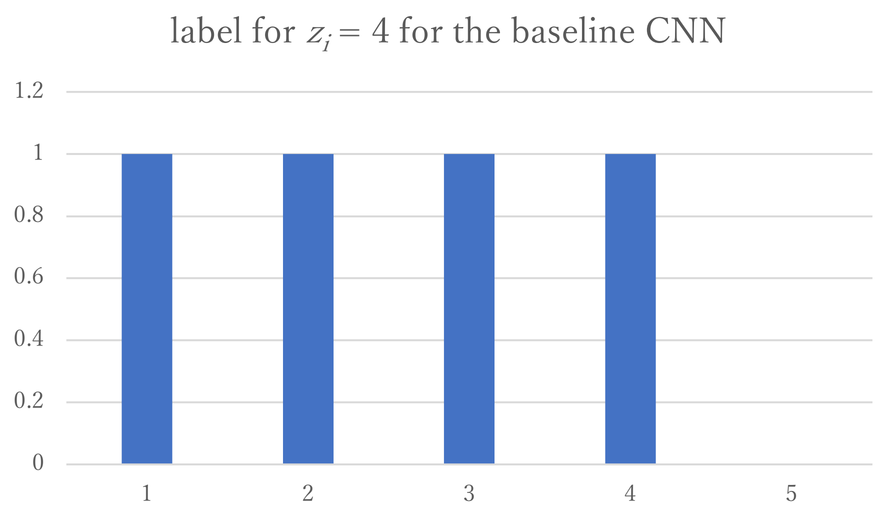

Figure S1C

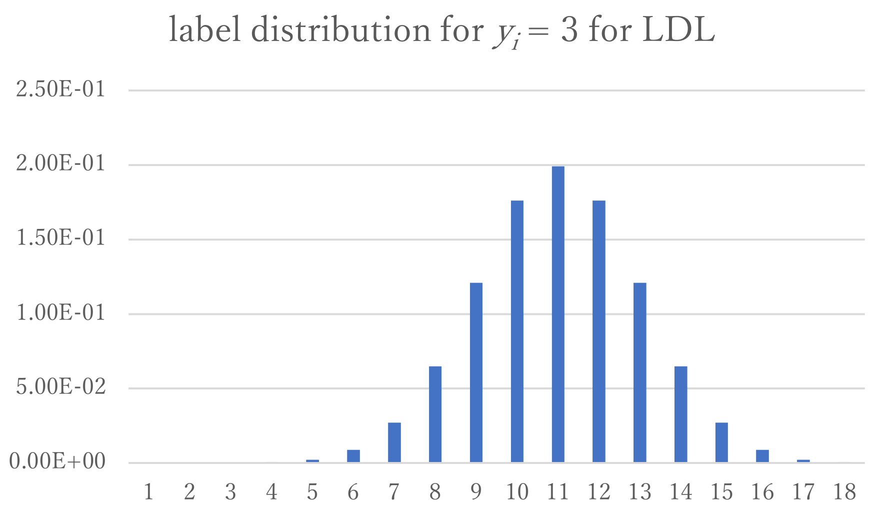

Figure S1D

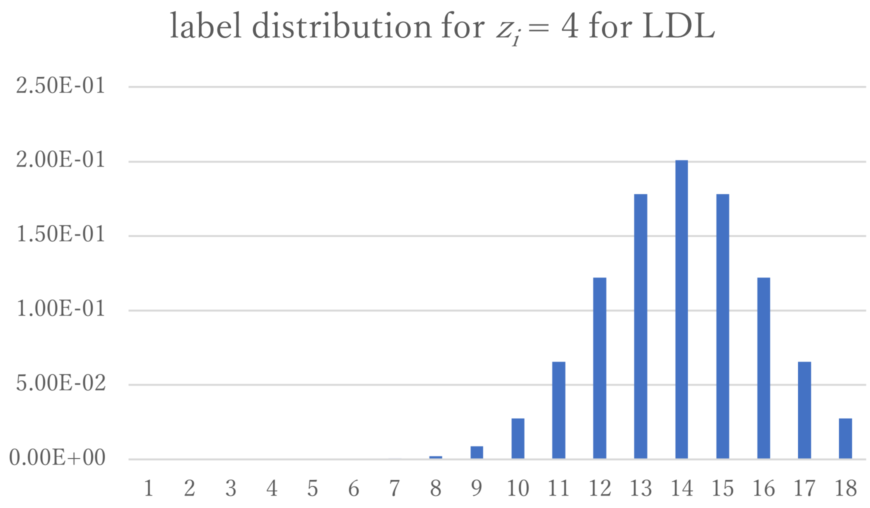

Supplementary Material S2

Results of the five-fold cross validation (CV) for the baseline CNN and the proposed CNN.

Tables 3-5 of the main text show the results of the CNN models when 5,160 WSIs from Radboud University Medical Center and 5,456 WSIs from Karolinska Institute were used as the development and unseen test sets, respectively.

In this Supplementary material 2, the two CNN models (the baseline CNN and the proposed CNN of EfficientNet B3 with LDL (D = 60)) were evaluated using 5-fold CV and the 10,616 WSI images (5,160 WSIs from Radboud University Medical Center and 5,456 WSIs from Karolinska Institute). It is speculated that many participants in the original PANDA challenge used 5-fold or 10-fold CV of the 10616 WSI images, for the development of their models.

The following Table S2 shows the results of 5-fold CV for the two CNN models. As shown in the Table S2, cross-validated QWK and cross-validated accuracy of the proposed CNN were better than those of the baseline CNN.

Table S2. Results of the five-fold cross validation of the baseline and the proposed CNNs.

CNN	Cross-validated QWK	Cross-validated accuracy
Baseline CNN	0.866	0.640
Proposed CNN of EfficientNet B3 with LDL (D = 60)	0.870	0.732

Note: The 10,616 WSI images (5,160 WSIs from Radboud University Medical Center and 5,456 WSIs from Karolinska Institute) were used for the 5-fold cross validation.
